# Supplementary material for: MetaQTL: a package of new computational methods for the meta-analysis of QTL mapping experiments
Source: BMC Bioinformatics. 2007 Feb 8;8:49. doi: 10.1186/1471-2105-8-49 (PMC1808479; doi:10.1186/1471-2105-8-49)
Supplement: Additional File 7 — MetaQTL Package : jar file and tutorial. This Zip archive contains both the MetaQTL JAR file and the files of the tutorial. [file 1471-2105-8-49-S7.zip › org.inra.metaqtl/doc/org/metaqtl/bio/entity/Ontology.html]

Ontology


|  |  |  |
| --- | --- | --- |
| **Overview** \| **Package** \| **Class** \| **Use** \| **Tree** \| **Deprecated** \| **Index** \| **Help** | |  |
| **PREV CLASS**   **NEXT CLASS** | **FRAMES**    **NO FRAMES**     **All Classes** |  |
| SUMMARY: NESTED \| FIELD \| CONSTR \| METHOD | DETAIL: FIELD \| CONSTR \| METHOD |  |


---


## org.metaqtl.bio.entity Class Ontology

```
java.lang.Object
  org.metaqtl.bio.entity.BioEntity
      org.metaqtl.bio.entity.Ontology
```

**All Implemented Interfaces:**: IBioAdaptable, IBioEntity, IBioOntology

---

``` public class Ontology extends BioEntity implements IBioOntology ```

---

**Field Summary**

**Fields inherited from class org.metaqtl.bio.entity.BioEntity**

`name, parent, properties`


**Constructor Summary**

`Ontology()`


| **Method Summary** | |
| --- | --- |
| `IBioAdapter` | `getBioAdapter()` |
| `java.lang.String` | `getFunction()`             Returns the function of the ontology |
| `IBioOntologyTerm` | `getRoot()`             Returns the root of the ontology tree. |
| `IBioOntologyTerm` | `getTerm(IBioOntologyTerm term)`             Returns if exists the term in the ontology which matches the given one. |
| `IBioOntologyTerm` | `getTerm(java.lang.String termName)`             This method looks into the ontology if there is a term with the given name and returns it. |
| `int` | `getType()`             There are 2 main class of entities. |
| `void` | `setFunction(java.lang.String function)`             Set the function of the ontology. |
| `void` | `setRoot(IBioOntologyTerm root)`             Sets the root of the ontology. |
| `void` | `setRoot(OntologyTerm root)` |

**Methods inherited from class org.metaqtl.bio.entity.BioEntity**

`getName, getParent, getProperties, newBioEntity, setName, setProperties`

**Methods inherited from class java.lang.Object**

`clone, equals, finalize, getClass, hashCode, notify, notifyAll, toString, wait, wait, wait`

**Methods inherited from interface org.metaqtl.bio.IBioEntity**

`getName, getParent, getProperties, setName, setProperties`

**Constructor Detail**

### Ontology

```
public Ontology()
```


**Method Detail**

### setFunction

```
public void setFunction(java.lang.String function)
```

:   Set the function of the ontology.

    :   **Parameters:**: `function` -

---


### getFunction

```
public java.lang.String getFunction()
```

:   **Description copied from interface: `IBioOntology`**
:   Returns the function of the ontology

    :   **Specified by:**: `getFunction` in interface `IBioOntology`
    :   **Returns:**

---


### getRoot

```
public IBioOntologyTerm getRoot()
```

:   **Description copied from interface: `IBioOntology`**
:   Returns the root of the ontology tree.

    :   **Specified by:**: `getRoot` in interface `IBioOntology`
    :   **Returns:**: the root of the ontology tree.

---


### getTerm

```
public IBioOntologyTerm getTerm(IBioOntologyTerm term)
```

:   **Description copied from interface: `IBioOntology`**
:   Returns if exists the term in the ontology which
    matches the given one.

    :   **Specified by:**: `getTerm` in interface `IBioOntology`
    :   **Parameters:**: `term` - the term to look for in the ontology. **Returns:**: the term if found, null otherwise.

---


### getType

```
public int getType()
```

:   **Description copied from interface: `IBioEntity`**
:   There are 2 main class of entities. The first one deals with population
    biological entity, i.e population itself and individuals. The second class
    is a representation of microscopic biological entity from genome container
    to alleles.

    :   **Specified by:**: `getType` in interface `IBioEntity` **Specified by:**: `getType` in class `BioEntity`

---


### getBioAdapter

```
public IBioAdapter getBioAdapter()
```

:   **Specified by:**: `getBioAdapter` in interface `IBioAdaptable`

---


### setRoot

```
public void setRoot(IBioOntologyTerm root)
```

:   **Description copied from interface: `IBioOntology`**
:   Sets the root of the ontology.

    :   **Specified by:**: `setRoot` in interface `IBioOntology`
    :   **Parameters:**: `root` - the root of the ontology

---


### setRoot

```
public void setRoot(OntologyTerm root)
```

:   **Parameters:**: `root` - the root of the ontology.

---


### getTerm

```
public IBioOntologyTerm getTerm(java.lang.String termName)
```

:   **Description copied from interface: `IBioOntology`**
:   This method looks into the ontology if there is
    a term with the given name and returns it.

    :   **Specified by:**: `getTerm` in interface `IBioOntology`
    :   **Parameters:**: `termName` - the name of the term to find. **Returns:**: the term or null if not found.


---


|  |  |  |
| --- | --- | --- |
| **Overview** \| **Package** \| **Class** \| **Use** \| **Tree** \| **Deprecated** \| **Index** \| **Help** | |  |
| **PREV CLASS**   **NEXT CLASS** | **FRAMES**    **NO FRAMES**     **All Classes** |  |
| SUMMARY: NESTED \| FIELD \| CONSTR \| METHOD | DETAIL: FIELD \| CONSTR \| METHOD |  |


---
